# Exploring the Nutritional and Antimicrobial Properties of Wild Fruit, *Castanopsis tribuloides*: In Vitro and In Silico Insights for Potential Antimicrobial Drug Development

**DOI:** 10.1155/sci5/2106755

**Published:** 2025-06-17

**Authors:** Hmingremhlua Sailo, Laldinliana Khiangte, Lalremliani, Laldinfeli Ralte, Sagolshem Priyokumar Singh, Y. Tunginba Singh

**Affiliations:** ^1^Department of Botany, Mizoram University, Aizawl 796004, Mizoram, India; ^2^Department of Botany, Asufii Christian Institute, Mao 795150, Manipur, India; ^3^Department of Life Sciences (Botany), Manipur University, Imphal 795003, Manipur, India

**Keywords:** antibiotics, bioactive compounds, *C. tribuloides*, MD simulation, molecular docking

## Abstract

A wild edible fruit*, Castanopsis tribuloides*, has been scientifically evaluated for its antibacterial capability and nutritional composition. In vitro analysis showed it possesses antimicrobial properties against *Bacillus subtilis*, *Escherichia coli*, *Pseudomonas aeruginosa*, and *Staphylococcus aureus*. In silico analysis revealed that out of the identified 27 compounds isolated, 1-phenylbicyclo (3.2.2) nona-6,8-dien-2-one demonstrated a solid adherence to the Lipinski rule of drug design and exhibited a strong binding affinity to antibacterial enzymes tyrosyl-tRNA synthase (−8.13 kcal/mol) and dihydropteroate synthase (−8.84 kcal/mol). The protein–ligand complex revealed with MD simulation showed low RMSD (< 1 nm), higher SASA (170–180 nm^2^), and more consistent hydrogen bonding (∼1.2 bonds/frame). The binding energy evaluated using gmx_MMPBSA also yields a favorable total binding free energy for dihydropteroate synthase (Δ*G*_bind_ = −17.92 ± 0.84 kcal/mol) and tyrosyl-tRNA synthase (Δ*G*_bind_ = −12.75 ± 1.46 kcal/mol), indicating stable complex formation. In addition, the nuts are rich in various nutrients, such as carbohydrates (31.2 ± 0.08), proteins (20.51 ± 0.1 mg/g), dietary fiber (5.41 ± 0.01%), vitamin C (86.3 ± 0.26 mg/100 gm), and vitamin E (4.76 ± 0.02 mg/100 gm). The nuts also demonstrated significant antioxidant activity (52.25 ± 0.02 μg/mL), with a high amount of total phenolic (76.83 ± 0.02 mg QE/g) and flavonoid (70.4 ± 0.21 mg QE/g) content. These findings indicate the importance of *C. tribuloides* nuts as a valuable antibacterial and health-promoting resource.

## 1. Introduction

Fruits and nuts play a crucial role in the human diet by providing a variety of essential nutrients for growth and development [1]. Edible seeds and nuts are popular because of their abundant bioactive components, vital fatty acids, vitamins, amino acids, and essential minerals [2]. Indigenous groups have learned that exploring wild edible plants can significantly improve their nutritional intake. Recently, more attention has been paid to these medicinal plants, and it was found that their medicinal value lies in various bioactive phytochemical compounds present in the plant. Therefore, the knowledge of different compounds present in plants provides information to back up many ancient lore medicines which could be incorporated into modern health warfare [3].

In addition to their nutritional value, many of these wild plants possess untapped medicinal properties, offering potential solutions in the face of increasing antibiotic resistance, a major global health threat. Antibiotic resistance occurs when bacteria or other pathogens undergo genetic changes that allow them to withstand conventional treatments, resulting in infections that are difficult to eliminate [4]. The increasing prevalence of drug-resistant infections highlights the immediate requirement for innovative medications [5] and the examination of bioactive chemicals in wild edible fruits and seeds, which could potentially lead to the development of new therapeutic medicines.

Owing to the extensive historical use of therapeutic herbs in traditional medicine, there is an increasing demand to verify scientifically their nutritional and medicinal characteristics [6]. Molecular docking is a crucial technique in contemporary drug discovery to predict potential interactions between bioactive compounds and target proteins. Molecular dynamics (MD) simulations are critical computational methodologies in pharmacological discovery. They forecast the binding of small compounds to target proteins and the durability of these interactions over time. This process assists in the development of novel medicinal drugs [7].

The genus *Castanopsis*, a perennial member of the Fagaceae family, is well known for its edible seeds, which are rich in essential nutrients such as proteins, fats, and minerals, contributing to their significance as a food source. Beyond their nutritional value, these plants also play an important ecological role in forest ecosystems by supporting biodiversity and stabilizing soil structures. Among its species, *Castanopsis tribuloides* A. DC. (*C. tribuloides*) is native to Northeast India, Southeast Asia, and the Himalayan regions [8]. This species is also commonly found in Mizoram, India, where it often grows along roadsides, which are collected by locals and sold in the markets for considerably cheap prices. *C. tribuloides* also has a long history of traditional medicinal uses, where the leaves and bark are employed to treat headaches, diarrhea, and other ailments [9]. The leaves' paste is applied to relieve stomachaches and headaches, while the resin is used for diarrhea treatment. The bark is also known to alleviate chest discomfort [10]. In addition to its medicinal value, the nuts serve as a vital food source for both humans and wildlife, including bears, monkeys, hoolock gibbons, wild pigs, barking deer, porcupines, hog-badgers, squirrels, and various birds such as gray treepies, parakeets, red jungle fowls, kalij pheasants, and partridges [11]. Such diverse nutritional, ecological, and medicinal attributes make *C. tribuloides* a promising candidate for further pharmacological and nutritional studies [12].

However, the nutritional and medicinal properties of *C. tribuloides* nuts remain unexplored. This study, therefore, aimed to investigate the nutritional composition and evaluate the therapeutic potential of wild *C. tribuloides* nuts through in vitro and in silico approaches, with the ultimate goal of identifying potentially novel antimicrobial drug candidates.

## 2. Materials and Methods

### 2.1. Sample Collection

**Figure 1:**
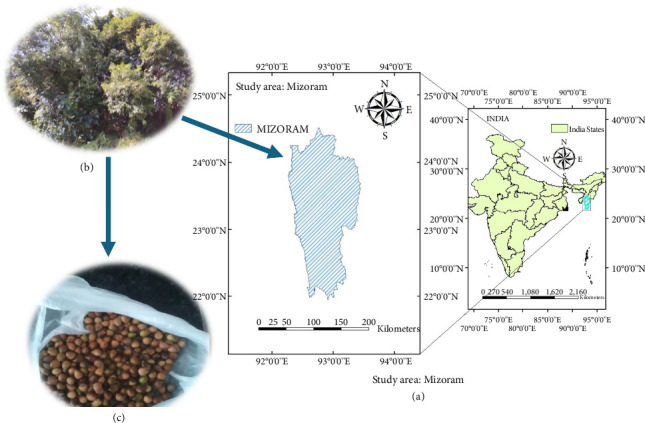
(a) Sample collection site, (b) photograph of the whole plant, and (c) nuts of *C. tribuloides*.

Nuts of *C. tribuloides* were obtained from Mizoram, India (Figure 1). The samples were taken to the department of botany, Mizoram University, for analysis. The obtained specimen (MZUH000052) was identified using existing literature [13, 14]. It was deposited at the department of botany, Mizoram University's herbarium.

### 2.2. Nutritional Properties

#### 2.2.1. Estimation of Carbohydrates, Protein Content, and Dietary Fiber

The total protein content was estimated by using the Lowry method [15]. For the estimation of protein, the extract was dissolved in 2% Na_2_Co_3_, 0.1 N NaOH, 1% nak tartarate, and 0.5% CuSO_4_, 1-part Folin-phenol (2N): 1-part water. The absorbance was measured at 660 nm, and a graph was plotted against the BSA working standard. The carbohydrate content was quantified using the anthrone method [16]. The total fiber content was estimated by using a phosphate buffer and heat-stable-amylase solution [17].

#### 2.2.2. Estimation of Vitamins

Vitamin C estimation was performed using ascorbic acid as standard and dissolving the samples in metaphosphoric acid, 10% acetic acid, 2,4-dinitrophenylhydrazine (DNPH), and cold 85% sulfuric acid (5 mL) solution; the optical density was measured at 521 nm [18]. For the estimation of vitamin E, Trolox (an analog of Vit. E) was used as a standard, and the sample was dissolved in anhydrous ethanol (0.5 mL), xylene (2 mL), bathophenanthroline (0.25 mL), FeCl_3_ (0.25 mL), and H_3_PO_4_ solution (0.25 mL). The absorbance was measured at 539 nm [19]. For vitamin K estimation, the standard Vit K1 was used following the Verma protocol [20]. In brief, the sample was mixed with 10% methanolic potassium hydroxide (or sodium methoxide) (10 mL) and methanol. The sample was filtered, and the absorbance was measured at 550 nm. For estimation of vitamin B6, pyridoxine chloride (Vit. B6) was used as a standard, and the sample was mixed with 5% trichloroacetic acid (TCA) (20 mL), diazotized p-nitroaniline (5 mM) (1.5 mL) reagent, CTAB (1 mM), and 0.1 N sodium carbonate (3 mL). The absorbance was measured at 480 nm [21]. To estimate vitamin A [22], sample treatment, reflux, UV exposure, and xylene extraction were performed, and the absorbance was measured at 335 nm.

#### 2.2.3. Essential Element Estimates

The nuts were dried in an oven at 60°C for 48 h and crushed into a fine powder. The fine powder (0.5 g) was digested with nitric acid (HNO_3_) and hydrogen peroxide (H_2_O_2_) in a 1:4 ratio [23], and the trace elements present were assayed using atomic absorption spectroscopy (AAS) (Shimadzu AA-7000).

#### 2.2.4. Fourier-Transform Infrared Spectroscopy (FTIR) Analysis

The dried nuts were ground to a fine powder form, and the functional groups were identified using FTIR (Shimadzu IRAffinity-1S, Japan) [24].

### 2.3. Phenol, Flavonoid Content, and 2,2-Diphenyl-Picrylhydrazyl (DPPH) Analysis

The nuts were dried and crushed into a fine powder. The phytochemical compounds in the sample were extracted with methanol using a Soxhlet apparatus. Folin–Ciocalteu reagent was used to determine the total phenolic content [25]. The extract was dissolved in Folin's reagent (0.2 mL) and sodium bicarbonate (5 mL), and the absorbance was measured at 560 nm. Total phenol was expressed as gallic acid equivalents (mg/g of the extracted component). The calorimetric method [26] was used to measure the flavonoid content. The extract was dissolved in 5% sodium nitrate (300 μL), aluminum chloride (300 μL), and 1 M NaOH (2 mL), and the absorbance was measured at 510 nm. The total flavonoid content was quercetin equivalent (mg/g of the isolated component). The antioxidant potential was determined using the DPPH radical scavenging protocol [27]. The extract was taken in different test tubes and made up to 1 mL using methanol. Next, 2 mL of DPPH was added to each test tube, and absorbance was measured at 520 nm. The percentage of DPPH was calculated using the following equation: % radical scavenging = ([abs control − abssample]/abscontrol) × 100. Ascorbic Acid was used as a standard.

### 2.4. *In Vitro* Analysis

#### 2.4.1. Antimicrobial Activity

The antibacterial activity of the methanol extract was determined using the agar well diffusion method. Two-gram negative bacteria, *Escherichia coli* (*E. coli*) (ATCC11229) and *Pseudomonas aeruginosa* (*P. aeruginosa*) (ATCC9027), along with two-gram positive bacteria, *Bacillus subtilis* (*B. subtilis*) (ATCC11774) and *Staphylococcus aureus* (*S. aureus*) (ATCC6908), were used. Ofloxacin (OFL) was used as a control, and the inhibition zones were measured in millimeters (mm) using an antibiotic inhibition scale [28].

#### 2.4.2. Minimum Inhibitory Concentration (MIC)

The methanol extract was prepared at different concentrations using the serial dilution method. The bacterial suspension (10.5 × 10^8^ CFU/mL), extract, and Mueller–Hinton broth (MHB) were added to each well of 96-well plates to measure the MIC. The plates were incubated for 24 h at 37°C, and resazurin was then added to each well and incubated for 3 h. A hue that shifts from blue (oxidized state) to pink (reduced state) served as a sign of bacterial development. The lowest concentration of the treatment that prevented the color change was then determined as the MIC [29].

### 2.5. GC–MS Analysis

The compounds present in the nuts' extract were identified using a GC–MS (Clarus 690, PerkinElmer, USA) (Autosystem XL) equipped with a capillary column (123.5 m × 678 m) coupled to a mass detector TurboMass Gold and PerkinElmer TurboMass 5.1 spectrometers with an Elite-1 (100% dimethylpolysiloxane), 123.5 m × 678 m, of the capillary column (123.5 m × 678 m). The National Institute of Standards and Technology (NIST 17) online library, Ver. 2.3, was used to compare and match the spectra of volatile chemicals discovered by GC–MS [30].

### 2.6. In Silico Analysis

#### 2.6.1. Drug Design and Drug-likeness

Compounds identified from GC–MS were first predicted based on Lipinski's rule of five for drug-likeness using https://www.swissadme.ch/.

#### 2.6.2. Molecular Docking

Then, the three-dimensional (3D) structures of tyrosyl-tRNA synthetase (TYRS) (Protein Data Bank [PDB] ID: 1JIJ) and dihydropteroate synthase (DHPS) (PDB ID: 3TYE) were obtained from the PDB (https://www.rcsb.org/) for potential antimicrobial drug design. The chemical structures of the chosen bioactive compounds 1-phenylbicyclo (3.2.2) nona-6,8-dien-2-one (PHY) (CID: 13734758) and the control OFL (CID: 4583) were obtained from the NCBI PubChem database (https://pubchem.nlm.nih.gov/), as shown in Table 1. Molecular docking was performed using AutoDock Vina software, and the docked result was visualized using Discovery Studio Visualizer [31].

#### 2.6.3. MD Simulation and gmx_MMPBSA

MD simulations were performed to evaluate the interaction strength and stability of the receptor–ligand complex. Topology files for small molecules were created using SOBTOP, whereas the protein topology was obtained using GROMACS commands. The Amber14sb force field and SPC water model were utilized to replicate physiological conditions, ensuring a 20 buffer between protein atoms and the edge of the water box. The system was neutralized using 0.15 mol/L saline [27]. Before the simulations, energy minimization was performed using 50,000 steepest-descent iterations. The system was equilibrated using NVT and NPT ensembles for 100 ps with a 2 fs timestep. A 100 ns MD simulation was subsequently conducted at 310 K. Trajectory analysis was conducted following the removal of periodic boundary conditions, and the final structure was superimposed onto the initial complex to assess interactions. Key measures, including root mean square deviation (RMSD), root mean fluctuation, number of hydrogen bonds (Hbs), and solvent accessible surface area (SASA) were analyzed. The binding free energy was computed using the gmx_MMPBSA [31].

### 2.7. Quality Control, Quality Assurance, and Statistical Analysis

This study instituted rigorous quality control measures to guarantee precision and dependability. Analytical-grade chemicals (HiMedia and Sigma) and ultrapure water were utilized throughout the process to minimize contamination hazards. Each sample was analyzed in triplicate (*n* = 3) to verify linearity, yielding low relative standard deviations (SDs) of ±0.72%–1.95%, which signifies good precision. The methanol extract was performed because it is widely used in scientific research due to its broad solvent range, high extraction efficiency, low boiling point, cost-effectiveness, and compatibility with various analytical techniques. Its ability to dissolve multiple compounds, penetrate cell walls, and extract intracellular components makes it ideal for obtaining comprehensive extract profiles. In addition, methanol extract's antimicrobial properties, ease of handling, and widespread use in phytochemical research contribute to its popularity as an extraction solvent. Data analysis was conducted using IBM SPSS Statistics Version 25 and GraphPad Prism. Statistical analysis was employed to validate the accuracy and significance of the measured concentrations of the nutrients such as trace elements, phenol, and flavonoid content. This ensures that the observed variations are meaningful and not due to random fluctuations, thereby strengthening the conclusions of the study. Chemical structures were created using Schrödinger Maestro 2023, and the binding site was predicted using Discovery Studio 2021. Docking and postdocking analyses were performed using AutoDock Tools 1.5.7. MD simulation and gmx_MMPBSA were performed using GROMACS 2024.3.

## 3. Results and Discussion

### 3.1. Carbohydrates, Proteins, and Total Dietary Fiber Content

**Figure 2:**
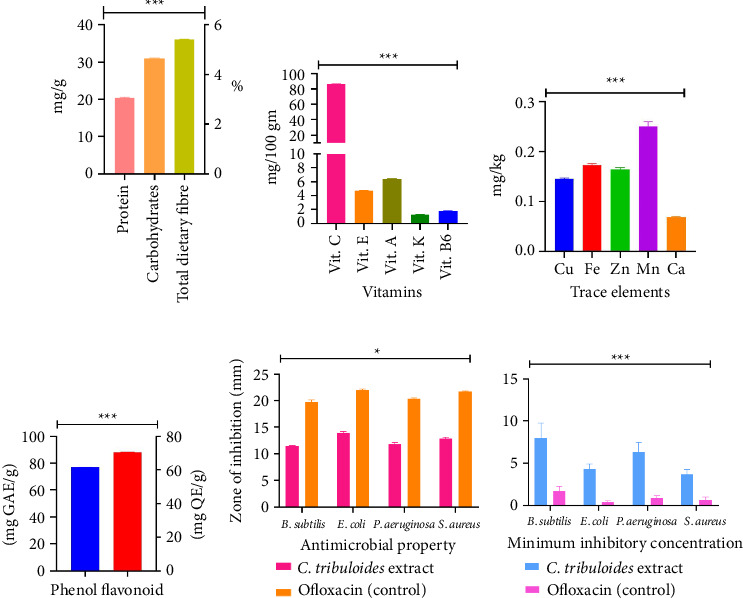
Nutritional composition showing (a) protein, carbohydrates, and total dietary fiber content; (b) vitamin profile; (c) trace element composition; (d) phenolic and flavonoid content; (e) antimicrobial properties of *C. tribuloides'* extract compared with the control (ofloxacin); and (f) minimum inhibitory concentration (MIC) of *C. tribuloides'* extract against selected bacterial strains in comparison to the control.

The analysis of *C. tribuloides* nuts revealed significant macronutrient content, with carbohydrate averaging at 31.2 ± 0.1 mg/g (Figure 2(a) and Supporting Table 1), protein at 20.51 ± 0.1 mg/g, and the total dietary fiber at 5.41 ± 0.01%. These findings revealed significant (*p* < 0.05) amounts of carbohydrates, protein, and dietary fiber. Carbohydrates are among the most important macronutrients for maintaining good health and fitness. They are high in energy and assist in regulating blood glucose and the breakdown of fatty acids. The all-around purpose is to incorporate enough carbohydrates into our diet [32]. Proteins obtained from plants are low-fat, low-calorie, and contain phytochemicals and fiber, essential in the human diet [33]. The substantial level of carbohydrates, protein, and total fiber in *C. tribuloides* nuts suggested they could provide essential macronutrients.

### 3.2. Concentration of Different Vitamins

The vitamin profile of *C. tribuloides* fruit, presented in Figure 2(b) and detailed in Supporting Table 1, indicates the concentration (*p* < 0.05) (Supporting Table 2) of different vitamins. Vitamin C (86.3 ± 0.26 mg/100 g) had the highest concentration, while vitamin K (1.24 ± 0.01 mg/100 g) had the lowest concentration. The study found many vitamins C, E, A, K, and B6. Vitamin C prevents scurvy and improves overall health [34]. Vitamin E is a lipid-soluble component of the cell's antioxidant defense mechanism that can only be absorbed through food. Vitamin E has been demonstrated to be beneficial against oxidation, which has been connected to various ailments and diseases, including cancer, aging, arthritis, and cataracts [35]. Vitamin A is a necessary nutrient in the human diet. It originates from foods high in beta-carotene, composed of two retinol molecules [36]. Vitamin K refers to a class of fat-soluble vitamins. They are regarded as necessary cofactors for creating numerous proteins involved in coagulation and calcium homeostasis in humans [37]. Vitamin B6 also plays a role in fatty acid biosynthesis, the breakdown of certain storage compounds in animals and plants, and the biosynthesis of plant hormones, neurotransmitters, and organelle-specific compounds [38]. The high vitamin content underscores their potential as a nutritious source of broad health benefits.

### 3.3. Composition of Essential Elements

The elemental composition, as illustrated in Figure 2(c) and detailed in Supporting Table 1, revealed relative quantities of several essential elements. Of the elements analyzed, Mn had the highest concentration at 0.24 mg/kg, while Ca had the lowest concentration at 0.06 mg/kg. Supporting Table 4 (one-way ANOVA) indicates a statistically significant difference (*p* < 0.05) in the concentrations of these trace elements. These elements are essential for various metabolic processes. Fe can regulate lipid content and the formation of red blood cells in the body. Other elements, such as Zn, are essential in enzymes and catalytic functions. While Mn, Ca, and Cu are necessary for growth development in plants and animals [39].

### 3.4. Phenol, Flavonoid Content, and DPPH Activity

The extract of *C. tribuloides* nuts exhibited high levels of total phenolic (76.83 ± 0.02 mg GAE/g) and flavonoid content (70.4 ± 0.21 mg QE/g), as depicted in Figure 2(d). Antioxidant potential, measured through DPPH activity, showed a strong scavenging effect with an IC_50_ of 52.29 ± 0.01 μg/mL (Supporting Table 1). The *t*-test (Supporting Table 5) revealed a significant difference (*p* < 0.05) in the total phenol and flavonoid content. Phenols yield substantial benefits for humans because they are antioxidants and can also break down free radicals in the body. Flavonoids have been found to have anticancer and antioxidant properties [40]. Previous studies have also reported that plant antioxidant properties and phytochemical contents are highly correlated [41]. Our study showed that wild *C. tribuloides* is a rich source of phenols and flavonoids with high antioxidant activity, underscoring its potential as an antioxidant source [41].

### 3.5. Antimicrobial Activity and MIC

The antimicrobial activity of the methanolic extract evaluated by the agar well diffusion method and MIC assessments demonstrated significant (*p* < 0.05) (Supporting Table 6) activity against four pathogenic bacterial strains as detailed in Supporting Table 3 and illustrated in Figures 2(e) and 2(f). The antimicrobial activity of the extract had reduced efficacy relative to OFL (control); however, it still displayed significant (*p* < 0.05) antibacterial activity. Among the evaluated pathogens, *E. coli* exhibited the highest susceptibility to the extract, demonstrating a zone of inhibition (13.67 ± 0.34 mm), signifying considerable antibacterial efficacy. The extract also exhibited significant inhibition against *S. aureus* (12.45 ± 0.12 mm) and *B. subtilis* (11.57 ± 0.57 mm). *P. aeruginosa*, however. demonstrated the least susceptibility to the extract (11.27 ± 0.10 mm). A zone of inhibition measuring 12–18 mm is typically regarded as a potential antimicrobial for plant extracts or natural products, especially when tested against resistant bacterial strains [42]. Therefore, despite the extract inhibitory zones of the extract being inferior to those of OFL, its efficacy nevertheless underscores strong antibacterial potential.

The MIC values further emphasized the significant antimicrobial potential (Supporting Table 7) of the extract of *C. tribuloides* as shown in Figure 2(f). The extract exhibited the highest efficacy against *B. subtilis* (7.33 ± 0.07 mg/mL) among the evaluated pathogens, followed by *P. aeruginosa* (6.67 ± 0.69 mg/mL) and *E. coli* (5.47 ± 0.13 mg/mL). *S. aureus* (4.33 ± 0.27 mg/mL) necessitated the highest concentration for inhibition. For plant extracts or natural compounds, MIC values ranging from 4 to 8 mg/mL may indicate potential for further enhancement [43]. Therefore, despite the MIC values of the extract exceeding those of OFL, it exhibited antibacterial efficacy especially against *B. subtilis* and *P. aeruginosa*, highlighting its potential as a viable alternative antimicrobial agent [44]. These findings also underscore the considerable antibacterial efficacy of the extract, especially against *E. coli*, a notable pathogen in gastrointestinal and urinary tract infections [45].

The observed activity may also be attributed to the phytochemical composition of the extract, notably its elevated levels of phenols and flavonoids, recognized for their efficacy against several microbial pathogens [46]. These bioactive molecules offer a promising pathway for developing alternative medicines, particularly considering increasing antibiotic resistance. The results underscore the therapeutic potential of *C. tribuloides* extracts in addressing bacterial infections, especially those induced by multidrug-resistant strains such as *P. aeruginosa* [47]. Additional research and refinement may improve its effectiveness for wider clinical use. The study emphasizes these bacterial species due to their clinical significance and the pressing necessity for new antimicrobial medicines to tackle public health issues. The in silico analysis further investigates the extract's bioactive components, providing a mechanistic comprehension of their effects. These findings highlight the extract's potential as a source of novel antimicrobial agents, facilitating its application in disease treatment and pharmaceutical development.

### 3.6. Pearson Correlation and Regression Analysis

**Figure 3:**
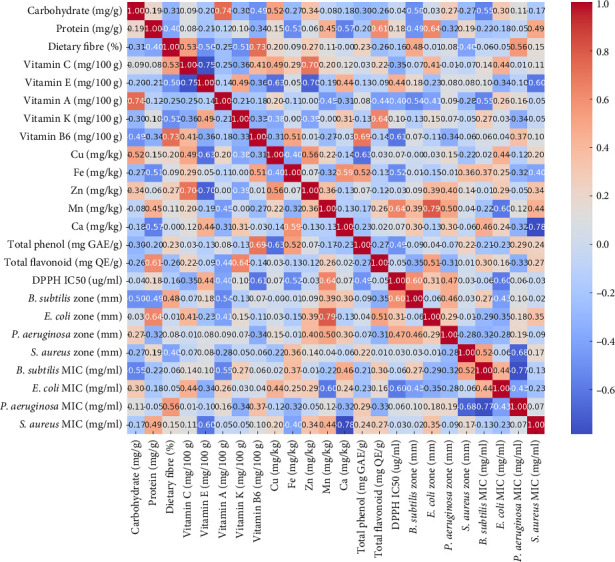
Heatmap showing Pearson correlation of various nutrients and their effects.

The dataset consists mainly of numerical measurements with presumed linear relationships, rendering Pearson correlation as shown in Figure 3. The total phenol concentration and flavonoid content demonstrated a robust positive correlation (0.89), underscoring their collective contribution to antioxidant activity. The total flavonoid content is positively connected to its antioxidant activity (0.64), indicating its substantial role in free radical scavenging. The phenol content was positively associated with the *E. coli* inhibition zone (0.64), suggesting a potential association between elevated phenol levels and improved antibacterial capabilities. Dietary fiber positively correlated with Mn (0.64), indicating a possible association between fiber content and trace element concentrations. Cu exhibited a moderate positive connection with total phenol content (0.52), indicating its contribution to the enhancement of phytochemical production. Mn positively correlated with phenolic content (0.48) and protein content (0.45), reinforcing its significance in synthesizing bioactive compounds. Finally, vitamin B6 showed a robust positive connection with dietary fiber (0.73), signifying a synergistic interaction in their nutritional content.

The coefficient analysis (Table 2) for total phenol content is −0.5956, and it is statistically significant (*p*=0.047), suggesting that higher phenolic content is associated with inhibition zone (i.e., more significant antimicrobial activity). The coefficient for total flavonoid content is −0.8141, with a *p* value of 0.068, which is on the borderline of significance. This suggests a potential negative relationship with antimicrobial activity, but it is not statistically strong at the 5% significance level. The coefficient for Mn is −0.5991, with a *p* value of 0.075, indicating a near-significant negative effect on the inhibition zone, suggesting that higher manganese levels might enhance antimicrobial activity. Protein content, vitamin C, and dietary fiber have *p* values greater than 0.05, meaning they do not significantly contribute to explaining the inhibition in this model.

These findings emphasize the complex interplay between bioactive compounds and nutrients in influencing antioxidant and antimicrobial properties [48]. While phenolic and flavonoid contents emerged as significant contributors, the borderline significance of manganese and flavonoids in antimicrobial activity highlights the need for further investigation to confirm their roles.

### 3.7. Compound Prediction Using FTIR

**Figure 4:**
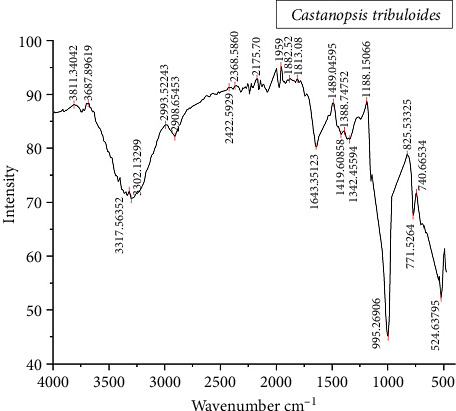
FTIR analysis of *C. tribuloides'* nut showing different peaks.

The FTIR spectra of the nuts, illustrated in Figure 4 and detailed in Supporting Table 8, showed maximum peaks between 500 cm^−1^ and 1500 cm^−1^, which showed that peaks were more abundant in the fingerprint region (500 cm^−1^ and 1500 cm^−1^) in comparison to the functional group region (1500 cm^−1^ and 4000 cm^−1^) [49]. No prominent peak was observed between the regions 2220 and 2260 cm^−1^, indicating the absence of cyanide groups in the plant extract [49]. The peaks (as shown in Supporting Table 1) predict the presence of cellulose, protein, phosphate, lipids, aromatic, amines, aliphatic amine, alkene, alkane, and lignin [50–55], suggesting diverse medicinal properties [56].

FTIR is an analytical technique that uses high resolution to identify chemical and structural components of various plant samples. The FTIR spectrum of *C. tribuloides* nuts showed various peaks, indicating the presence of multiple compounds that could contribute to improving human health. These findings provide a basis for further exploration of the medicinal and therapeutic potential of *C. tribuloides* [57].

### 3.8. Compound Isolation Using GC–MS

**Figure 5:**
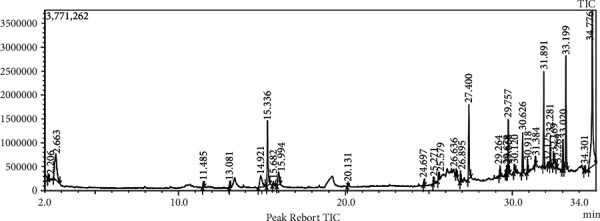
GC–MS chromatogram of methanolic extract of *C. tribuloides*.

GC–MS analysis of *C. tribuloides* nut's extract identified 27 compounds, as depicted in the chromatogram (Figure 5). Among these, 13 compounds are beneficial for various purposes (Supporting Table 9). Compounds such as hydroquinone, 1,2,4-oxadiazole, 3-(3,5 bistrifluoromethyl)phenyl, hexadecanoic acid, methyl ester, docosane, hexanedioic acid, and bis(2-ethylhexyl) were reported to have antimicrobial and antifungal potentials [58]. Other compounds, such as acetic acid, 2-ethylhexyl ester, were also reported to have various commercial uses (resins, lacquers, and nitrocellulose), while hexatriacontane was also noted for its antidepressant properties [59]. Hydroxytoluene, methyl stearate, n-hexadecanoic acid, and 9-octadecenamide (Z) reported anti-inflammatory and antioxidant properties [60]. The identified compounds indicated that *C. tribuloides* has a wide range of therapeutic and economic potential [58–62].

### 3.9. Compound Test for Drug-Likeness

As determined by Lipinski's rule (Table 3), drug viability indicated that among the 27 compounds, PHY demonstrates the most favorable outcomes and was compared against the standard OFL. PHY has a molecular weight of 220.35 g/mol and exhibits moderate lipophilicity (log *p* value: 4.24). In comparison, OFL, with a molecular weight of 361.37 g/mol, demonstrated lower lipophilicity (log *p* value: 1.15) and higher polar surface area (75.01 Å), which resulted in its notably increased solubility in water. In terms of pharmacokinetic characteristics, PHY had significant gastrointestinal absorption and the ability to cross the blood–brain barrier, unlike OFL, which may not penetrate the blood–brain barrier. In addition, PHY also exhibited inhibitory effects on cytochrome P450 2D6. However, OFL did not demonstrate any inhibitory effects on the investigated cytochrome P450 enzymes. Both compounds complied with Lipinski's rule of five, demonstrating favorable drug-like properties [63].

### 3.10. In Silico Antibacterial Analysis Using Molecular Docking

**Figure 6:**
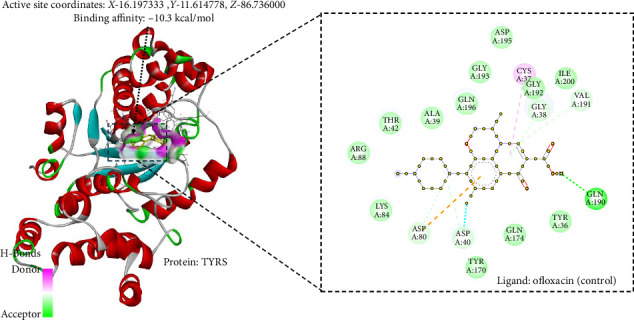
Ofloxacin (control) interaction with bacterial enzyme tyrosyl-tRNA synthase.

**Figure 7:**
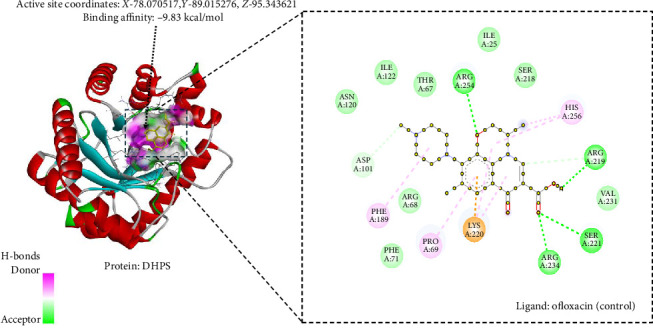
Ofloxacin (control) with bacterial enzyme dihydropteroate synthase.

**Figure 8:**
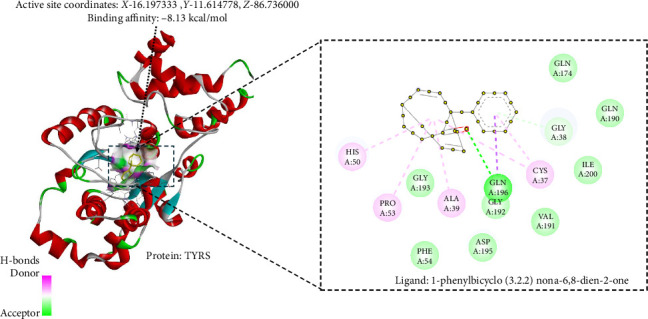
1-Phenylbicyclo (3.2.2) nona-6,8-dien-2-one interaction with bacterial enzyme tyrosyl-tRNA synthase.

**Figure 9:**
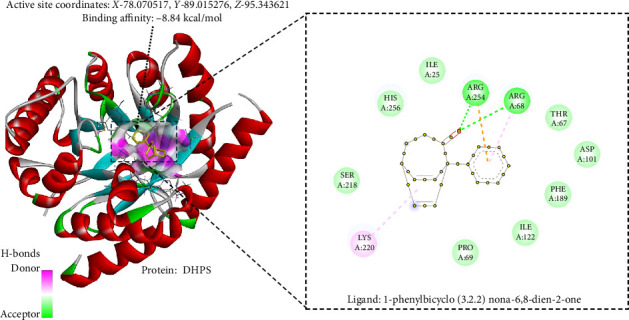
1-Phenylbicyclo (3.2.2) nona-6,8-dien-2-one interaction with bacterial enzyme dihydropteroate synthase.

The study conducted molecular docking (Table 4) of two bacterial enzymes, TYRS and DHPS, utilizing the ligands OFL (control) (Figures 6 and 7) and PHY (Figures 8 and 9). Against TYRS, the drug score of OFL is 0.84, with a binding affinity of −10.3 kcal/mol, ligand efficiency of −0.54, and an inhibition constant (KI) of 54 nM. The ligand PHY exhibited a drug score of 0.82, binding affinity of −8.13 kcal/mol, ligand efficiency of −0.43, and inhibition constant (KI) of 19.97 nM. While PHY showed a weaker binding energy than OFL, its lower KI value suggests stronger inhibitory effects.

For the enzyme DHPS, OFL demonstrated a stronger binding energy of −9.83 kcal/mol, a ligand efficiency of −0.48, and an inhibition constant (KI) of 61.7 nM with a 0.81 drug score. The ligand PHY achieved a drug score of 0.81, binding affinity of −8.84 kcal/mol, ligand efficiency of 0.43, and inhibition constant (KI) of 10.8 nM, indicating it to be a potential ligand for developing antibiotics that target DHPS. Despite having weaker binding energy, PHY again demonstrated a lower KI value, indicating potential as a promising ligand for developing antibiotics targeting DHPS and TYRS [64].

TYRS are essential enzymes involved in protein synthesis. Its primary function is to catalyze the attachment of tyrosine to its respective tRNA molecule. TYRS is a crucial target for developing antimicrobial drugs because it plays a vital role in translating genetic information into proteins. Bacterial TYRS has distinct structural and functional characteristics compared to its eukaryotic counterparts, which scientists have utilized to develop specific inhibitors. These inhibitors can interfere with bacterial protein synthesis, reducing the growth or death of bacterial cells [65]. DHPS is another crucial enzyme involved in folate production in bacteria. This facilitates the chemical reaction between p-aminobenzoic acid (PABA) and p-hydroxybenzoate, forming dihydropteroate. DHPS is a critical target for antibiotics because it plays a significant role in DNA synthesis and cell development, which are essential processes in the body. Sulfonamide antibiotics, which are structural analogs of PABA, hinder the activity of DHPS and consequently impede folate production, resulting in the inhibition or death of bacteria [66]. A docking binding free energy lower than −5 kcal/mol has a high binding affinity and is regarded as having potential for drug development. The binding score of *C. tribuloides* compound, PHY against TYRS (−8.13 kcal/mol), as shown in Figure 6 and DHPS (−8.84 kcal/mol) as shown in Figure 7, may exhibit dual antibacterial potential [67].

### 3.11. Analysis of a Dynamic Complex of Protein and Ligand Using MD Simulation

**Figure 10:**
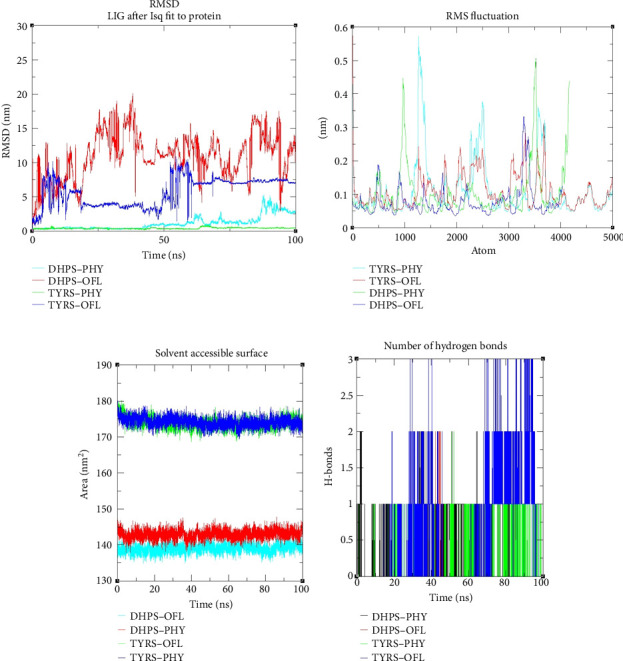
(a) RMSD, (b) RMSF, (c) hydrogen bonds, and (d) SASA of ligand (PHY) and control (OFL) with bacterial enzymes (DHPS and TYRS).

MD simulation was conducted to further investigate the structural stability, dynamic behavior, and interaction mechanisms of biomolecular complexes between the proteins: TYRS and DHPS and the ligand: PHY and OFL under physiological conditions. The RMSD plot shows the structural stability of four ligand–protein complexes over a 100 ns simulation period (Figure 10(a)). The DHPS–PHY and TYRS–PHY complexes exhibit minimal fluctuations, maintaining low RMSD values (∼1-2 nm), indicating stable interactions. Conversely, DHPS–OFL displays significant instability with RMSD values fluctuating widely between 5 and 20 nm, suggesting major structural deviations. TYRS–OFL demonstrates moderate stability with RMSD values stabilizing around 5 nm after an initial fluctuation. Overall, the DHPS–OFL complex shows the highest instability, while the PHY complex (both DHPS and TYRS) appears to be the most stable [68].

The root mean square fluctuation (RMSF) plot reveals the flexibility of different regions in the four ligand–protein complexes (Figure 10(b)). The TYRS–PHY and DHPS–PHY systems exhibit higher fluctuations in certain regions, reaching peaks above 0.4 nm, indicating flexible loop regions or terminal residues. The TYRS–OFL and DHPS–OFL systems demonstrate relatively lower fluctuations throughout, suggesting more stable protein structures. Notably, the PHY systems exhibit distinct peaks in specific regions, possibly reflecting dynamic behavior critical for ligand interactions. Overall, the PHY systems show more pronounced flexibility in key regions, while the OFL systems maintain relatively restrained movements [68].

The number of Hbs' analysis further supports TYRS–PHY as the optimal candidate (Figure 10(c)). The TYRS–OFL system demonstrates the highest number of Hbs, often maintaining values between 1.5 and 2.5, suggesting strong and consistent ligand–protein interactions. TYRS–PHY also shows a stable Hb (1-2) throughout the simulation. Conversely, DHPS–PHY and DHPS–OFL display minimal Hb interactions, with frequent drops to zero, indicating weaker and less stable binding. Considering Hb along with the earlier RMSD and RMSF analyses, TYRS–PHY emerges as the best candidate, combining stable structural behavior, controlled flexibility, and consistent Hb interactions [68].

The SASA analysis provides further insights into the structural characteristics of the complex (Figure 10(d)). The TYRS–PHY and TYRS–OFL systems maintain higher SASA values, consistently around 175–180 nm^2^, suggesting better solvent exposure and potential for favorable interactions in aqueous environments. Conversely, the DHPS–PHY and DHPS–OFL systems show lower SASA values, stabilizing around 140–145 nm^2^, indicating reduced solvent exposure and potentially less favorable binding dynamics. Given that TYRS–PHY not only demonstrates stable SASA but also exhibits superior stability, controlled flexibility, and consistent Hb in previous analyses, TYRS–PHY continues to stand out as the optimal candidate [69].

Both complexes demonstrated binding affinities and structural interactions with their targets, confirming PHY's potential as an antimicrobial agent. The TYRS–PHY complex stands out as a more promising candidate due to its superior stability (RMSD < 1 nm), higher solvent accessibility (SASA ∼175–180 nm^2^), consistent Hb (∼1.2 bonds/frame), and stronger binding free energy (−75.4 kJ/mol). In contrast, the DHPS–PHY complex, with higher RMSD and lower SASA, exhibited a weaker profile but remains a viable candidate for an antimicrobial candidate based on the MD simulation of the complex [69].

### 3.12. Principal Component Analysis (PCA) of TYRS–PHY and DHPS–PHY Complexes

**Figure 11:**
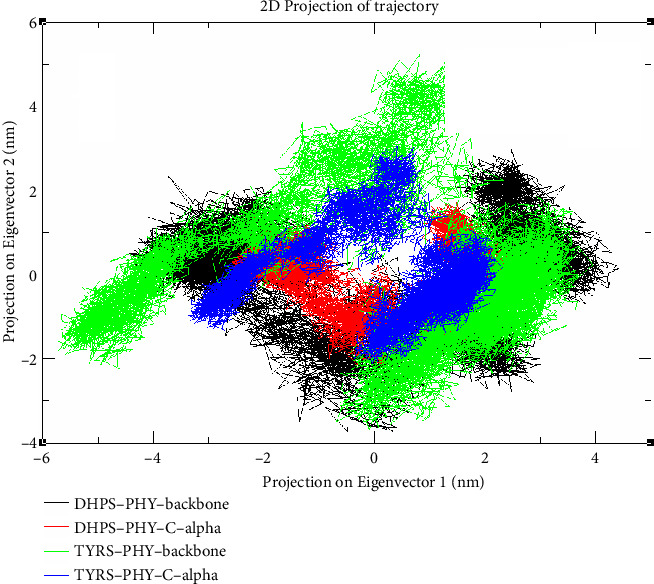
2D projection of molecular dynamics trajectories for TYRS–PHY and DHPS–PHY complexes on principal components.

The two-dimensional projection of MD trajectories onto the initial two eigenvectors illustrated in Figure 11 reveals unique conformational regions for the TYRS–PHY and DHPS–PHY complexes, as determined by backbone and C-alpha components. The TYRS–PHY–C-alpha group constitutes a dense cluster, with data points spanning roughly −2 to +2 nm along Eigenvector 1 and −1 to +2 nm along Eigenvector 2, signifying its structural stability. Conversely, the DHPS–PHY–C-alpha group has a broader distribution, with data points ranging from −4 to +4 nm on Eigenvector 1 and −3 to +3 nm on Eigenvector 2, indicating more flexibility. TYRS–PHY and DHPS–PHY backbone trajectories have unique, albeit overlapping distributions, highlighting differences in dynamic behavior. These projections elucidate the structural dynamics of the complexes, which are essential for comprehending ligand-binding mechanisms in antimicrobial docking investigations. TYRS–PHY complexes' compact and stable conformational states indicate an enhanced capacity for establishing robust and consistent contacts with ligands, as evidenced by docking simulations that revealed decreased RMSD values, increased Hb stability, and advantageous binding energies for TYRS. These discoveries are essential for enhancing antimicrobial candidates aimed at bacterial enzymes such as TYRS and DHPS, hence aiding in developing more potent inhibitors [70, 71].

### 3.13. Analysis of Binding Interaction of Protein and Ligand Using gmx_MMPBSA

**Figure 12:**
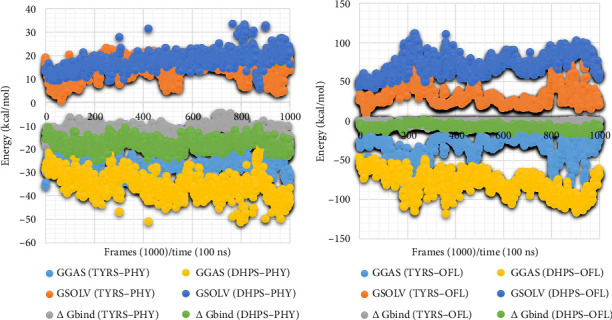
Comparison of energy component contributions for (a) DHPS–PHY and TYRS–PHY and (b) TYRS–OFL and DHPS–OFL complexes using gmx_MMPBSA.

Finally, the gmx_MMPBSA analysis was conducted to calculate the binding free energy between the protein (DHPS and TYRS) and its ligand (PHY and OFL) in MD simulations. This method combines the molecular mechanics (MM) force field with the Poisson–Boltzmann or generalized Born solvation model to estimate the energetics of complex formation. The binding interactions between TYRS and PHY were evaluated using gmx_MMPBSA, as shown in Figure 12(a), and revealed a favorable total binding free energy (Δ*G*_bind_ = −12.75 ± 1.46 kcal/mol), suggesting stable complex formation under the simulated conditions. This binding affinity was primarily driven by van der Waals interactions (ΔVDWAALS = −19.55 ± 0.54 kcal/mol) and electrostatics (ΔEEL = −7.51 ± 1.21 kcal/mol), partially counteracted by polar solvation effects (ΔEGB = 16.84 ± 0.58 kcal/mol). In addition, nonpolar solvation (ΔESURF = −2.53 ± 0.09 kcal/mol) contributed favorably to the overall binding energy. These results highlight that the TYRS–PHY complex achieves stable binding primarily through hydrophobic and electrostatic interactions, supporting its potential in biochemical and pharmacological research.

Similarly, the binding interaction between DHPS and PHY was assessed using gmx_MMPBSA, as shown in Figure 12(a). The analysis demonstrated a more favorable total binding free energy (Δ*G*_bind_ = −17.92 ± 0.84 kcal/mol), indicating even stronger complex stability. This enhanced stability was largely driven by van der Waals interactions (ΔVDWAALS = −24.61 kcal/mol) and electrostatics (ΔEEL = −10.87 ± 0.55 kcal/mol), partially offset by polar solvation effects (ΔEGB = 21.02 ± 0.18 kcal/mol). Nonpolar solvation (ΔESURF = −3.46 ± 0.06 kcal/mol) also positively contributed to the binding energy.

In comparison, the control complexes with OFL showed higher energy profiles (Figure 12(b)). The TYRS–OFL complex displayed a total binding free energy of −10.54 ± 1.32 kcal/mol, while the DHPS–OFL complex exhibited a total binding free energy of −15.43 ± 1.12 kcal/mol. PHY complexes exhibited stronger binding affinities than their OFL counterparts. The TYRS–PHY complex showed a more favorable binding energy than TYRS–OFL, while DHPS–PHY displayed a stronger binding affinity than DHPS–OFL. These results indicate that PHY may outperform OFL in terms of binding stability, reinforcing its potential as an effective antimicrobial candidate [72, 73].

## 4. Conclusion

*C. tribuloides* nuts were found to have significant nutritional and medicinal potential. Nutritional value includes high levels of carbohydrates (31.2 mg/g), proteins (20.51 mg/g), dietary fiber (5.41%), and vital vitamins, including vitamins C (86.3 mg/100 g) and E (4.76 mg/100 g). These nutrients provide a significantly enhanced nutritious diet. Elevated concentrations of phenolics (76.83 mg GAE/g) and flavonoids (70.4 mg QE/g) enhance antioxidant efficacy. At the same time, notable antimicrobial activity against bacterial strains, including *E. coli* (13.67 mm inhibition zone) and *S. aureus* (12.45 mm inhibition zone), underscores their therapeutic potential. Molecular docking revealed PHY as a potential option for targeting the bacterial enzymes TYRS (−8.13 kcal/mol) and DHPS (−8.84 kcal/mol). MD simulations demonstrated significant stability of the complex, corroborated by persistent Hb and advantageous total binding energy. *C. tribuloides* nuts represent a promising nutritional supply and a unique repository of antibacterial chemicals, with potential applications in functional foods and pharmaceuticals.

## Figures and Tables

**Table 1:** Summary of ligand and enzyme used in the study.

S. No	Name of ligand	Chemical structure	Molecular formula	Molecular weight (g/mol)	PubChem ID
1	1-Phenylbicyclo (3.2.2) nona-6,8-dien-2-one	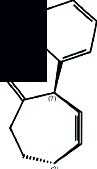	C_15_H_14_O	210.27 g/mol	13734758
2	Ofloxacin	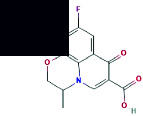	C_18_H_20_FN_3_O_4_	361.4 g/mol	4583

**S. No**	**Name of enzyme**	**Chemical structure**	**Structural insight**		**RCSB ID**

1	TYRS	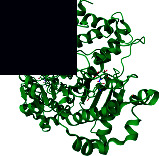	Crystal structure of *S. aureus* TYRS in complex with SB-239629		1JIJ
2	DHPS	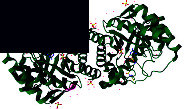	Dihydropteroate synthase in complex with DHP–STZ		3TYE

**Table 2:** Regression analysis for antimicrobial activity predictors.

Variable	Coefficient	Std err	*t*-Statistic	*p* value	Confidence interval (lower)	Confidence interval (upper)
Intercept	59.7295	30.5	1.958	0.189	−71.502	190.961
Total phenol	−0.5956	0.133	−4.469	0.047	−1.169	−0.022
Total flavonoid	−0.8141	0.223	−3.645	0.068	−1.775	0.147
Protein	0.3902	0.22	1.776	0.218	−0.555	1.335
Dietary fiber	0.021	0.096	0.219	0.847	−0.391	0.433
Vitamin C	0.5685	0.326	1.744	0.223	−0.834	1.971
Mn	−0.5991	0.174	−3.443	0.075	−1.348	0.15
Cu	−0.2445	0.178	−1.371	0.304	−1.012	0.523

**Table 3:** Molecular properties of the bioactive compounds: physicochemical properties and pharmacokinetics.

Physicochemical properties		
Compounds	1-Phenylbicyclo (3.2.2) nona-6,8-dien-2-one	Ofloxacin
Molecular formula	C_15_H_24_O	C_16_H_19_N_3_O_4_S
Molecular weight	220.35 g/mol	361.37 g/mol
Rotatable bonds	2	2
H-bond acceptor bonds	1	6
H-bond donor atoms	1	1
Molar refractivity	71.97	101.83
Polar surface area	20.23 Å	75.01 Å
Lipophilicity (consensus)	4.24	1.15
Water solubility	Moderately soluble	Soluble

**Pharmacokinetics**		

Gastrointestinal absorption	High	High
Blood–brain barrier permeation	Yes	No
P-glycoprotein substrate	No	Yes
Cytochrome P450 1A2 inhibitor	No	No
Cytochrome P450 2D6 inhibitor	Yes	No
Cytochrome P450 3A4 inhibitor	No	No
Drug-likeness (Lipinski rule)	Yes	Yes

**Table 4:** Docking result showing comparative drug–ligand interactions of receptors and ligands.

Receptor	Ligand	Drug score	Binding energy (kcal/mol)	Ligand efficiency	Inhibition constant^d^ (KI) (nM)
TYRS	Ofloxacin	0.84	−10.3	−0.54	54
	1-Phenylbicyclo (3.2.2) nona-6,8-dien-2-one	0.82	−8.13	−0.43	19.97

DHPS	Ofloxacin	0.82	−9.83	−0.48	61.7
	1-Phenylbicyclo (3.2.2) nona-6,8-dien-2-one	0.81	−8.84	−0.43	10.8

## Data Availability

Data will be made available on request.

## Supporting Information

Supplementary file contains tables of nutritional, phytochemicals, antimicrobial profile, functional groups, and metabolic compounds present in *C. tribuloides.*

Supplementary Table 1: Nutritional, phytochemical, and antimicrobial profile of *C. tribuloides*.

Supplementary Table 2: One-way ANOVA analysis of protein, carbohydrates, and dietary content in *C. tribuloides'* seed.

Supplementary Table 3: One-way ANOVA analysis of vitamin content in *C. tribuloides'* seed.

Supplementary Table 4: One-way ANOVA analysis of trace elements' concentration in *C. tribuloides'* seed.

Supplementary Table 5: The *t*-test analysis of protein, phenol, and flavonoid content in *C. tribuloides'* seed.

Supplementary Table 6: One-way ANOVA analysis of antimicrobial activities of *C. tribuloides'* seed.

Supplementary Table 7: One-way ANOVA analysis of minimum inhibitory concentration of *C. tribuloides'* seed.

Supplementary Table 8: FTIR spectrum with functional groups present in *C. tribuloides'* seed.

Supplementary Table 9: Compounds identified in the methanolic extract of *C. tribuloides'* seed using gas chromatography.

## References

[1] Alt K. W., Al-Ahmad A., Woelber J. P. (2022). Nutrition and Health in Human Evolution-Past to Present. *Nutrients*.

[2] Moreda-Piñeiro J., Herbello-Hermelo P., Domínguez-González R., Bermejo-Barrera P., Moreda-Piñeiro A. (2016). Bioavailability Assessment of Essential and Toxic Metals in Edible Nuts and Seeds. *Food Chemistry*.

[3] Seal T. (2011). Determination of Nutritive Value, Mineral Contents and Antioxidant Activity of Some Wild Edible Plants From Meghalaya State, India. *Asian Journal of Applied Sciences*.

[4] Frieri M., Kumar K., Boutin A. (2017). Antibiotic Resistance. *Journal of Infection and Public Health*.

[5] McCullough A. R., Parekh S., Rathbone J., Del Mar C. B., Hoffmann T. C. (2016). A Systematic Review of the Public’s Knowledge and Beliefs About Antibiotic Resistance. *Journal of Antimicrobial Chemotherapy*.

[6] Chaudhary R. P. (2000). Forest Conservation and Environmental Management in Nepal: A Review. *Biodiversity & Conservation*.

[7] Li X. M., Li M. T., Jiang N. (2021). Network Pharmacology-Based Approach to Investigate the Molecular Targets of Sinomenine for Treating Breast Cancer. *Cancer Management and Research*.

[8] Chen P., Lian J. Y., Wu B., Cao H. L., Li Z. H., Wang Z. F. (2023). Draft Genome of *Castanopsis chinensis*, A Dominant Species Safeguarding Biodiversity in Subtropical Broadleaved Evergreen Forests. *BMC Genomic Data*.

[9] Joshi K., Joshi R., Joshi A. R. (2011). Indigenous Knowledge and Uses of Medicinal Plants in Macchegaun, Nepal. *Indian Journal of Traditional Knowledge*.

[10] Dolai N., Karmakar I., Kumar R. B., Bala A., Mazumder U. K., Haldar P. K. (2012). Antitumor Potential of *Castanopsis indica* (Roxb. Ex Lindl.) A. DC. Leaf Extract Against Ehrlich’s Ascites Carcinoma Cell. *Indian Journal of Experimental Biology*.

[11] Malla B., Chhetri R. B. (2009). Indigenous Knowledge on Ethnobotanical Plants of Kavrepalanchowk District. *Kathmandu University Journal of Science, Engineering and Technology*.

[12] Tuyen P. T., Khang D. T., Minh L. T. (2016). Phenolic Compounds and Antioxidant Activity of *Castanopsis phuthoensis* and *Castanopsis grandicicatricata*. *International Letters of Natural Sciences*.

[13] Sawmliana M. (2003). *The Book of Mizoram Plants*.

[14] Singh N. P., Singh K. P., Singh D. K. (2022). *Flora of Mizoram*.

[15] Waterborg J. H. (2009). The Lowry Method for Protein Quantitation. *The Protein Protocols Handbook*.

[16] Buckan D. S. (2015). Estimation of Glycemic Carbohydrates From Commonly Consumed Foods Using Modified Anthrone Method. *Indian Journal of Applied Research*.

[17] McCleary B. V., Sloane N., Draga A. (2015). Determination of Total Dietary Fibre and Available Carbohydrates: A Rapid Integrated Procedure That Simulates In Vivo Digestion. *Starch*.

[18] Desai A. P., Desai S. (2019). UV Spectroscopic Method for Determination of Vitamin C (Ascorbic Acid) Content in Different Fruits in South Gujarat Region. *International Journal of Environmental Sciences & Natural Resources*.

[19] Rutkowski M., Grzegorczyk K. (2007). Modifications of Spectrophotometric Methods for Antioxidative Vitamins Determination Convenient in Analytic Practice. *Acta Scientiarum Polonorum Technologia Alimentaria*.

[20] Verma H. (2019). Simultaneous Estimation of Menaquinone-7 and Cholecalciferol in Combined Pharmaceutical Dosage Forms by Ultraviolet Spectrophotometry. *Asian Journal of Pharmaceutical Sciences*.

[21] Abdul Kadir A. N. (2010). Spectrophotometric Determination of Vitamin B6 by Coupling witH Diazotized P-Nitroaniline. *Rafidain Journal of Science*.

[22] Oteng P., Otchere J. K., Adusei S., Mensah R. Q., Tei-Mensah E. (2020). Vitamin Analysis, Trace Elements Content, and Their Extractabilities in *Tetrapleura tetraptera*. *Journal of Chemistry*.

[23] Singh R., Singh D. P., Kumar N., Bhargava S. K., Barman S. C. (2010). Accumulation and Translocation of Heavy Metals in Soil and Plants From Fly Ash Contaminated Area. *Journal of Environmental Biology*.

[24] Berthomieu C., Hienerwadel R. (2009). Fourier Transform Infrared (FTIR) Spectroscopy. *Photosynthesis Research*.

[25] McDonald S., Prenzler P. D., Antolovich M., Robards K. (2001). Phenolic Content and Antioxidant Activity of Olive Extracts. *Food Chemistry*.

[26] Chang C. C., Yang M. H., Wen H. M., Chern J. C. (2020). Estimation of Total Flavonoid Content in Propolis by Two Complementary Colometric Methods. *Journal of Food and Drug Analysis*.

[27] Baliyan S., Mukherjee R., Priyadarshini A. (2022). Determination of Antioxidants by DPPH Radical Scavenging Activity and Quantitative Phytochemical Analysis of *Ficus religiosa*. *Molecules*.

[28] Ertürk Ö. (2006). Antibacterial and Antifungal Activity of Ethanolic Extracts From Eleven Spice Plants. *Biologia*.

[29] Parvekar P., Palaskar J., Metgud S., Maria R., Dutta S. (2020). The Minimum Inhibitory Concentration (MIC) and Minimum Bactericidal Concentration (MBC) of Silver Nanoparticles Against *Staphylococcus aureus*. *Biomaterial Investigations in Dentistry*.

[30] Ralte L., Sailo H., Kumar N. S., Singh Y. T. (2024). Exploring the Pharmacological Potential of *Lepionurus sylvestris* Blume: From Folklore Medicinal Usage to Modern Drug Development Strategies Using In Vitro and In Silico Analyses. *BMC Complementary Medicine and Therapies*.

[31] Liu M., Wang Y., Deng W. (2024). Combining Network Pharmacology, Machine Learning, Molecular Docking and Molecular Dynamic to Explore the Mechanism of Chufeng Qingpi Decoction in Treating Schistosomiasis. *Frontiers in Cellular and Infection Microbiology*.

[32] Khowala S., Verma D., Banik S. P. (2008). *Biomolecules: (Introduction, Struction & Function)*.

[33] Hoffman J. R., Falvo M. J. (2004). Protein-Which is Best?. *Journal of Sports Science and Medicine*.

[34] Traber M. G., Stevens J. F. (2011). Vitamins C and E: Beneficial Effects From a Mechanistic Perspective. *Free Radical Biology and Medicine*.

[35] Rizvi S., Raza S. T., Ahmed F., Ahmad A., Abbas S., Mahdi F. (2014). The Role of Vitamin E in Human Health and Some Diseases. *Sultan Qaboos University Medical Journal*.

[36] Fragoso Y. D., Campos N. S., Tenrreiro B. F., Guillen F. J. (2012). Systematic Review of the Literature on Vitamin A and Memory. *Dementia & Neuropsychologia*.

[37] Schwalfenberg G. K. (2017). Vitamins K1 and K2: The Emerging Group of Vitamins Required for Human Health. *Journal of Nutrition and Metabolism*.

[38] Parra M., Stahl S., Hellmann H. (2018). Vitamin B6 and its Role in Cell Metabolism and Physiology. *Cells*.

[39] Sailo H., Ralte L., Hnamte R., Singh Y. T. (2023). The Assessment of Rice and Paddy Fields in Mizoram, India, Suggests a Need for Better Health Risk Management. *Water, Air, & Soil Pollution*.

[40] Shrestha P., Modi B., Aryal Surya P. (2020). Total Phenolic and Flavonoids Contents, Antioxidant Activities, Phytochemical and Nutritional Analysis of *Castanopsis indica* (Indian Chestnut). *Natural Resources and Sustainable Development*.

[41] Khang D. T., Dung T. N., Elzaawely A. A., Xuan T. D. (2016). Phenolic Profiles and Antioxidant Activity of Germinated Legumes. *Foods*.

[42] Murugaiyan J., Kumar P. A., Rao G. S. (2022). Progress in Alternative Strategies to Combat Antimicrobial Resistance: Focus on Antibiotics. *Antibiotics*.

[43] Nielsen T. R., Kuete V., Jäger A. K., Meyer J. J. M., Lall N. (2012). Antimicrobial Activity of Selected South African Medicinal Plants. *BMC Complementary and Alternative Medicine*.

[44] Mahadevi R., Salmen S. H., Alfarraj S., Wainwright M., Kavitha R. (2021). Screening and Characterization of Phytochemical Content of Methanolic Extract of Rhizome of *Curcuma amada* and Their Antibacterial Activity Against MRSA. *Applied Nanoscience*.

[45] Kpemissi M., Metowogo K., Melila M. (2020). Acute and Subchronic Oral Toxicity Assessments of *Combretum micranthum* (Combretaceae) in Wistar Rats. *Toxicology Reports*.

[46] Górniak I., Bartoszewski R., Króliczewski J. (2019). Comprehensive Review of Antimicrobial Activities of Plant Flavonoids. *Phytochemistry Reviews*.

[47] Horcajada J. P., Montero M., Oliver A. (2019). Epidemiology and Treatment of Multidrug-Resistant and Extensively Drug-Resistant *Pseudomonas aeruginosa* Infections. *Clinical Microbiology Reviews*.

[48] da Silva J. F. M., de Souza M. C., Matta S. R., de Andrade M. R., Vidal F. V. N. (2006). Correlation Analysis Between Phenolic Levels of Brazilian Propolis Extracts and Their Antimicrobial and Antioxidant Activities. *Food Chemistry*.

[49] Lingegowda D. C., Kumar J. K., Prasad A. D., Zarei M., Gopal S. (2012). FTIR Spectroscopic Studies on *Cleome gynandra*-Comparative Analysis of Functional Group Before and After Extraction. *Romanian Journal of Biophysics*.

[50] Trivedi M., Branton A., Trivedi D., Nayak G., Bairwa K., Jana K. (2015). Spectroscopic Characterisation of Disodium Hydrogen Orthophosphate and Sodium Nitrate After Biofield Treatment. *Journal of Chromatography & Separation Techniques*.

[51] Reig F. B., Adelantado J. G., Moreno M. M. (2002). FTIR Quantitative Analysis of Calcium Carbonate (Calcite) and Silica (Quartz) Mixtures Using the Constant Ratio Method. Application to Geological Samples. *Talanta*.

[52] Naumann D. (2000). Infrared Spectroscopy in Microbiology. *Encyclopedia of Analytical Chemistry*.

[53] Thakkar S. S., Thakor P., Doshi H., Ray A. (2017). 1,2,4-Triazole and 1,3,4-Oxadiazole Analogues: Synthesis, MO Studies, In Silico Molecular Docking Studies, Antimalarial as DHFR Inhibitor and Antimicrobial Activities. *Bioorganic & Medicinal Chemistry*.

[54] Baker M. J., Trevisan J., Bassan P. (2014). Using Fourier Transform IR Spectroscopy to Analyze Biological Materials. *Nature Protocols*.

[55] Mariswamy Y., Gnanaraj W. E., Antonisamy J. M. (2012). FTIR Spectroscopic Studies on *Aerva lanata* (L.) Juss. Ex Schult. *Asian Journal of Pharmaceutical and Clinical Research*.

[56] Maobe M. A., Nyarango R. M., Box P. O. (2013). Fourier Transformer Infra-red Spectrophotometer Analysis of *Urtica dioica* Medicinal Herb Used for the Treatment of Diabetes, Malaria and Pneumonia in Kisii Region, Southwest Kenya. *World Applied Sciences Journal*.

[57] Kumar J. K., Prasad A. D. (2011). Identification and Comparison of Biomolecules in Medicinal Plants of *Tephrosia tinctoria* and *Atylosia albicans* by Using FTIR. *Romanian Journal of Biophysics*.

[58] Jyoti M. A., Nam K. W., Jang W. S. (2016). Antimycobacterial Activity of Methanolic Plant Extract of *Artemisia capillaris* Containing Ursolic Acid and Hydroquinone Against *Mycobacterium tuberculosis*. *Journal of Infection and Chemotherapy*.

[59] Goel R. K., Gawande D. Y., Lagunin A. A., Poroikov V. V. (2018). Pharmacological Repositioning of *Achyranthes aspera* as an Antidepressant Using Pharmacoinformatic Tools PASS and PharmaExpert: A Case Study With Wet Lab Validation. *SAR and QSAR in Environmental Research*.

[60] Adnan M., Nazim Uddin Chy M., Mostafa Kamal A. T. (2019). Investigation of the Biological Activities and Characterization of Bioactive Constituents of *Ophiorrhiza rugosa* Var. Prostrata (D. Don) & Mondal Leaves Through In Vivo, In Vitro, and In Silico Approaches. *Molecules*.

[61] Aparna V., Dileep K. V., Mandal P. K., Karthe P., Sadasivan C., Haridas M. (2012). Anti-Inflammatory Property of N-Hexadecanoic Acid: Structural Evidence and Kinetic Assessment. *Chemical Biology & Drug Design*.

[62] Gumgumjee N. M., Hajar A. S. (2015). Antibacterial Activities and GC-MS Analysis of Phytocomponents of *Ehretia abyssinica* R. Br. Ex Fresen. *International Journal of Applied Biology and Pharmaceutical Technology*.

[63] Walters W. P. (2012). Going Further Than Lipinski’s Rule in Drug Design. *Expert Opinion on Drug Discovery*.

[64] Ralte L., Sailo H., Kumar R., Khiangte L., Kumar N. S., Singh Y. T. (2024). Identification of Novel AKT1 Inhibitors From *Sapria himalayana* Bioactive Compounds Using Structure-Based Virtual Screening and Molecular Dynamics Simulations. *BMC Complementary Medicine and Therapies*.

[65] Xiao Z. P., Ma T. W., Liao M. L. (2011). Tyrosyl-tRNA Synthetase Inhibitors as Antibacterial Agents: Synthesis, Molecular Docking and Structure-Activity Relationship Analysis of 3-Aryl-4-Arylaminofuran-2 (5H)-Ones. *European Journal of Medicinal Chemistry*.

[66] Fernández-Villa D., Aguilar M. R., Rojo L. (2019). Folic Acid Antagonists: Antimicrobial and Immunomodulating Mechanisms and Applications. *International Journal of Molecular Sciences*.

[67] da Fonseca A. M., Caluaco B. J., Madureira J. M. (2024). Screening of Potential Inhibitors Targeting the Main Protease Structure of SARS-CoV-2 via Molecular Docking, and Approach With Molecular Dynamics, RMSD, RMSF, H-Bond, SASA and MMGBSA. *Molecular Biotechnology*.

[68] Li D. D., Ma J. R., Huang Q. R., Man R. J., Zhao L. (2024). Exploring Protein-Berberine Interactions via Molecular Dynamics and MM/GBSA Calculations. *Journal of Molecular Liquids*.

[69] Cao X., Hummel M. H., Wang Y., Simmerling C., Coutsias E. A. (2024). Exact Analytical Algorithm for the Solvent-Accessible Surface Area and Derivatives in Implicit Solvent Molecular Simulations on GPUs. *Journal of Chemical Theory and Computation*.

[70] Imran M., Imran M., Haider A. (2023). Polyvinylpyrrolidone and Chitosan-Coated Magnetite (Fe_3_O_4_) Nanoparticles for Catalytic and Antimicrobial Activity With Molecular Docking Analysis. *Journal of Environmental Chemical Engineering*.

[71] Singh H., Raja A., Prakash A., Medhi B. (2023). Gmx_qk: An Automated Protein/Protein-Ligand Complex Simulation Workflow Bridged to MM/PBSA, Based on GROMACS and Zenity-Dependent GUI for Beginners in MD Simulation Study. *Journal of Chemical Information and Modeling*.

[72] Irfan I., Ali A., Ubaid A. (2024). Synergistic Antimicrobial Activity, MD Simulation Studies and Crystal Structure of Natural Alcohol Motif Containing Novel Substituted Cinnamates. *Journal of Biomolecular Structure and Dynamics*.

[73] Pisano M. B., Kumar A., Medda R. (2019). Antibacterial Activity and Molecular Docking Studies of a Selected Series of Hydroxy-3-Arylcoumarins. *Molecules*.

