# Supplementary material for: Exploring the Nutritional and Antimicrobial Properties of Wild Fruit, Castanopsis tribuloides: In Vitro and In Silico Insights for Potential Antimicrobial Drug Development
Source: Scientifica (Cairo). 2025 Jun 17;2025:2106755. doi: 10.1155/sci5/2106755 (PMC12187442; doi:10.1155/sci5/2106755)
Supplement: Supporting Information — Additional supporting information can be found online in the Supporting Information section. [file 2106755.f1.docx]

Supplementary TABLE 1: Nutritional, Phytochemical, and Antimicrobial Profile of *C. tribuloides*

| **S. No** | **Nutritional Content** | **Parameters** | ***C. tribuloides* Seed** |
| --- | --- | --- | --- |
| 1. |  | Carbohydrate content | 31.2±0.08 mg/g |
| 2. |  | Protein content | 20.51±0.1 mg/g |
| 3. |  | Total Dietary Fibre | 5.41±0.01 % |
| **S. No** | **Vitamin Content** | **Parameters** | ***C. tribuloides* Seed** |
| 1. |  | Vitamin C | 86.3±0.26 mg/100gm |
| 2. |  | Vitamin E | 4.76±0.02 mg/100gm |
| 3. |  | Vitamin A | 6.45±0.02 mg/100gm |
| 4. |  | Vitamin K | 1.24±0.01 mg/100gm |
| 5. |  | Vitamin B6 | 1.76±0.14 mg/100gm |
| **S. No** | **Trace elements** | **Parameters** | ***C. tribuloides* Seed** |
| 1. |  | Cu | 0.145±0.001 (mg/kg) |
| 2. |  | Fe | 0.173±0.002 (mg/kg) |
| 3. |  | Zn | 0.162±0.003 (mg/kg) |
| 4. |  | Mn | 0.244±0.009 (mg/kg) |
| 5. |  | Ca | 0.066±0.001 (mg/kg) |
| **S. No** | **Phytochemical content** | **Parameters** | ***C. tribuloides* Seed** |
| 1. |  | Total Phenol Content | 76.83±0.02 (mg GAE/g) |
| 2. |  | Total Flavonoid Content | 70.4±0.21 (mg QE/g) |
| 3. |  | DPPH (IC_50_) | 52.25±0.02 (ug/ml) |
| **S. No** | **Anti-microbial activities** | **Bacterial strain** | ***C. tribuloides* Seed** |
| 1. |  | *B. subtilis* | 11.57±0.57 (mm) |
| 2. |  | *E. coli* | 13.67±0.34 (mm) |
| 3. |  | *P. aeruginosa* | 11.27±0.1 (mm) |
| 4. |  | *S. aureus* | 12.45±0.12 (mm) |
| **S. No** | **Minimum Inhibitory Concentration** | **Bacterial strain** | ***C. tribuloides* Seed** |
| 1. |  | *B. subtilis* | 7.36 ± 0.02 (mg/ml) |
| 2. |  | *E. coli* | 5.47 ± 0.13 (mg/ml) |
| 3. |  | *P. aeruginosa* | 6.51 ± 0.09 (mg/ml) |
| 4. |  | *S. aureus* | 4.25 ± 0.03 (mg/ml) |

Supplementary TABLE 2: One-Way ANOVA analysis of protein carbohydrates and dietary content in *C. tribuloides* seed

| **ANOVA table** | **SS** | **DF** | **MS** | **F (DFn, DFd)** | **P value** |
| --- | --- | --- | --- | --- | --- |
| Treatment (between columns) | 993.5 | 2 | 496.8 | F (2, 6) = 27262 | P<0.0001 |
| Residual (within columns) | 0.1093 | 6 | 0.01822 |  |  |
| Total | 993.6 | 8 |  |  |  |

Supplementary TABLE 3: One-Way ANOVA analysis of vitamins content in *C. tribuloides* seed

| **ANOVA table** | **SS** | **DF** | **MS** | **F (DFn, DFd)** | **P value** |
| --- | --- | --- | --- | --- | --- |
| Treatment (between columns) | 16491 | 4 | 4123 | F (4, 10) = 96524 | P<0.0001 |
| Residual (within columns) | 0.4271 | 10 | 0.04271 |  |  |
| Total | 16492 | 14 |  |  |  |

Supplementary TABLE 4: One-Way ANOVA analysis of trace elements concentration in *C. tribuloides* seed

| **ANOVA table** | **SS** | **DF** | **MS** | **F (DFn, DFd)** | **P value** |
| --- | --- | --- | --- | --- | --- |
| Treatment (between columns) | 0.05064 | 4 | 0.01266 | F (4, 10) = 193.8 | P<0.0001 |
| Residual (within columns) | 0.0006533 | 10 | 6.533e-005 |  |  |
| Total | 0.05129 | 14 |  |  |  |

Supplementary TABLE 5: T-Test analysis of protein phenol and flavonoid content in *C. tribuloides* seed

| **T-test** | |
| --- | --- |
| P value | <0.0001 |
| P value summary | **** |
| Significantly different (P < 0.05)? | Yes |
| One- or two-tailed P value? | Two-tailed |
| t, df | t=28.86, df=4 |

Supplementary TABLE 6: One-Way ANOVA analysis of anti-microbial activities of *C. tribuloides* seed

| **ANOVA table** | **SS** | **DF** | **MS** | **F (DFn, DFd)** | **P value** |
| --- | --- | --- | --- | --- | --- |
| Treatment (between columns) | 8.623 | 3 | 2.874 | F (3, 8) = 86.17 | P<0.0001 |
| Residual (within columns) | 0.2669 | 8 | 0.03336 |  |  |
| Total | 8.890 | 11 |  |  |  |

Supplementary TABLE: 7 One-Way ANOVA analysis of Minimum Inhibitory Concentration of *C. tribuloides* seed

| **ANOVA table** | **SS** | **DF** | **MS** | **F (DFn, DFd)** | **P value** |
| --- | --- | --- | --- | --- | --- |
| Treatment (between columns) | 16.28 | 3 | 5.428 | F (3, 8) = 249.9 | P<0.0001 |
| Residual (within columns) | 0.1737 | 8 | 0.02172 |  |  |
| Total | 16.46 | 11 |  |  |  |

Supplementary TABLE 8: FTIR spectrum with functional group of present in *C. tribuloides* seed

| **S. No** | **Wave number (cm^-1^)** | **Functional group** |
| --- | --- | --- |
| 1 | 524.63 |  |
| 2 | 740.66 | -C-H |
| 3 | 771.52 |  |
| 4 | 825.53 | Na (NO_3_) |
| 5 | 995.26 |  |
| 6 | 1188.15 | C-O cellulose |
| 7 | 1324.55 | C-O |
| 8 | 1388.74 | CH aliphatic bending group |
| 9 | 1419.60 | stretching -C=O inorganic carbonate |
| 10 | 1489.04 | stretching -C=O inorganic carbonate |
| 11 | 1643.35 | Alkene |
| 12 | 1813.08 |  |
| 13 | 1882.52 |  |
| 14 | 1959 |  |
| 15 | 2175.70 | C=C conjugate and C triple C |
| 16 | 2368.65 | C=C conjugate and C triple C |
| 17 | 2422.59 | C=C conjugate and C triple C |
| 18 | 2908.65 | CH and CH2 stretching aliphatic group |
| 19 | 3302.13 | O-H acids |
| 20 | 3317.56 | N-H group |
| 21 | 3689.89 | N-H Amines |
| 22 | 3811.34 |  |

Supplementary TABLE 9: Compounds identified in the methanolic extract of  *C. tribuloides* seed using Gas-Chromatography.

| **SI. No** | **Name** | **Molecular formula** | **Molecular Weight** | **Retention Time** | **Area%** | **Height%** | **Uses** |
| --- | --- | --- | --- | --- | --- | --- | --- |
| 1. | Boric acid, trimethyl ester | C_3_H_9_BO_3_ | 104 | 2.663 | 8.00 | 3.06 |  |
| 2. | 1,3,5-Triazine-2,4,6-triamine | C_3_H_6_N_6_ | 189.13 | 11.485 | 0.51 | 0.70 |  |
| 3. | Acetic acid, 2-ethylhexyl ester | C_10_H_20_O_2_ | 172.26 | 13.081 | 0.42 | 0.76 | Resins, Lacquers, Nitrocellulose, Baking finishes. |
| 4. | Butylated Hydroxytoluene | C_15_H_24_O | 220.3 | 14.921 | 3.83 | 1.36 |  |
| 5. | Hydroquinone | C_6_H_6_O_2_ | 110.11 | 15.336 | 7.71 | 7.64 | Antimicrobial. |
| 6. | Butylated Hydroxytoluene | C_15_H_24_O | 220.35 | 15.994 | 2.72 | 1.54 | Anti-inflammatory, Antioxidant |
| 7. | Butane,1,4-bis(9,10-dihydro-9methyl anthra | C_34_H_34_ | 442 | 25.271 | 0.48 | 0.81 |  |
| 8. | 1,2,4-Oxadiazole, 3-(3,5bistrifluoromethylp | C_15_H_13_F_6_N_3_O_2_ | 381 | 25.579 | 0.67 | 0.87 | Antimicrobial, Antimalaria |
| 9. | 7,9-Di-tert-butyl-1-oxaspiro(4,5)deca-6,9-die | C_17_H_24_O_3_ | 276 | 26.636 | 0.47 | 0.74 |  |
| 10. | Hexadecanoic acid, methyl ester | C_17_H_34_O_2_ | 270.4 | 26.895 | 0.72 | 1.30 | Antifungal |
| 11. | n-Hexadecanoic acid | C_16_H_32_O_2_ | 256.42 | 27.400 | 7.04 | 8.71 | Antioxidant, Anti-inflamatory and Anti-carinogenic. |
| 12. | Docosane | CH_3_(CH_2_)_20_CH_3_ | 310.6 | 29.264 | 1.13 | 1.19 | Antifungal |
| 13. | Dotriacontane | C_32_H_66_ | 450.9 | 29.615 | 0.31 | 0.39 |  |
| 14. | Methyl stearate | C_19_H_38_O_2_ | 298.5 | 29.668 | 0.53 | 1.01 | Anti-  inflammatory,  Antihelmintic, Antinociceptive |
| 15. | 1-Isobutylsulfanylmethyl-2,8,9-trioxa-5-aza- | C_11_H_23_NO_3_SSi | 277.46 | 29.757 | 3.96 | 6.34 |  |
| 16. | Octadecanoic acid | C_18_H_36_O_2_ | 284.4 | 30.120 | 1.06 | 0.98 | Antiviral |
| 17. | Glutarimide, N-(2-octyl)- | C_13_H_23_O_2_ | 225.32 | 30.918 | 0.98 | 1.58 |  |
| 18. | 1-Cyclohexyldimethylsilyloxy-3,5-dimethylb | C_16_H_26_OSi | 262.46 | 31.384 | 0.51 | 0.92 |  |
| 19. | Eicosane | C_20_H_42_ | 282.2 | 31.891 | 7.37 | 11.29 |  |
| 20. | 2-Propenoic acid, 3-(4-methoxyphenyl) | C_10_H_10_O_3_ | 248.32 | 32.125 | 0.74 | 0.35 |  |
| 21. | 1-Cyclohexyldimethylsilyloxy-3,5-dimethylb | C_16_H_26_OSi | 262.46 | 32.281 | 2.39 | 3.64 |  |
| 22. | 1-Phenylbicyclo(3.2.2)nona-6,8-dien-2-one | C_15_H_14_O | 210.27 | 32.469 | 1.02 | 1.48 |  |
| 23. | 9-Octadecenamide, (Z)- | C_18_H_35_NO | 281.5 | 32.629 | 0.26 | 0.51 | Antioxidant |
| 24. | Hexanedioic acid, bis(2-ethylhexyl) ester | C_22_H_42_O_4_ | 370.5 | 33.020 | 2.84 | 3.52 | Antimicrobial |
| 25. | Hexatriacontane | C_36_H_74_ | 507.0 | 33.199 | 10.78 | 13.40 | Antidepressant |
| 26. | 2,3-Dihydroxypropyl icosanoate, 2TMS deri | C_29_H_62_O_4_SI_2_ | 386.6 | 34.301 | 0.49 | 0.61 |  |
| 27. | Dotriacontane | C_32_H_66_ | 450.9 | 34.776 | 27.46 | 18.36 | Antibacterial |
